# Complete Genome of *Bacillus thuringiensis* Myophage Spock

**DOI:** 10.1128/genomeA.00863-13

**Published:** 2013-12-05

**Authors:** Justin W. Maroun, Kelvin J. Whitcher, Karthik R. Chamakura, Gabriel F. Kuty Everett

**Affiliations:** Center for Phage Technology, Texas A&M University, College Station, Texas, USAa; Department of Biology, University of Mary Washington, Fredericksburg, Virginia, USAb

## Abstract

*Bacillus thuringiensis* is a Gram-positive, sporulating soil microbe with valuable pesticide-producing properties. The study of bacteriophages of *B. thuringiensis* could provide new biotechnological tools for the use of this bacterium. Here, we present the complete annotated genome of Spock, a myophage of *B. thuringiensis*, and describe its features.

## GENOME ANNOUNCEMENT

*Bacillus thuringiensis* is a Gram-positive, sporulating soil bacterium. It is an agriculturally important bacterium as it produces an endotoxin (the product of the *cry* gene) used in the biocontrol of insects. *B. thuringiensis* is grown in large quantities for the purpose of harvesting the Cry toxin. An estimated 83% of *B. thuringiensis* strains harbor temperate phages (1). Induction of these phages during batch growth causes lysis of the cultures and economic loss. The study of *B. thuringiensis* phages may give insight as to how to curb this problem. Here, we report the complete genome of the *B. thuringiensis* phage Spock.

Bacteriophage Spock was obtained from a soil sample collected in Harrisonburg, VA. Phage DNA was sequenced using 454 pyrosequencing at the Emory GRA Genome Center (Emory University, Atlanta, GA). Trimmed FLX titanium reads were assembled to a single contig at 208.9-fold coverage using the Newbler assembler, version 2.5.3 (454 Life Sciences), at default settings. PCR confirmed completed contigs. Genes were predicted using GeneMarkS (2) and corrected using software tools available on the Center for Phage Technology (CPT) Portal (https://cpt.tamu.edu/cpt-software/portal/). Transmission electron microscopy was performed at the University of Mary Washington.

Spock is a myophage with a unit genome of 161,497 bp and 280 unique coding sequences, of which 204 encode conserved hypothetical proteins and 20 encode novel hypothetical proteins. Spock has a coding density of 92.4% and GC content of 38.2%. The Spock TerL has homology to TerLs of phages with long terminal repeats. PAUSE (https://cpt.tamu.edu/cpt-software/releases/pause/) analysis of the raw sequencing data revealed that the long terminal repeat is 2,800 bp. The host range of Spock includes *B. anthracis* (delta Sterne) which lacks the virulence plasmids pXO1 and pXO2 that encode an exotoxin and the poly γ-glutamate capsule, respectively (3). The Spock genome is syntenic with the genomes of *B. cereus* phages B5S (JN797796.1) and B4 (NC_018863.1).

The genome encodes several proteins involved in DNA replication and recombination, including a DnaB-like helicase, DNA primase, DNA polymerase, dUTP pyrophosphatase, RecA, Holliday junction resolvase, and several DNA binding proteins. One HNH homing endonuclease was found. DNA and amino acid biosynthesis genes were identified, encoding ribonucleotide reductase subunits alpha and beta, deoxynucleotide monophosphate kinase, thymidylate synthase, dihydrofolate reductase, and *S*-adenosyl-l-methionine (SAM) methyltransferase. Several transcriptional regulators and sigma factors were identified as well. Spock also encodes a putative flavodoxin and a thioredoxin. Several structural components were identified (portal, prohead protease, capsid protein, tail sheath protein, an endo-beta-*N*-acetylglucosamidase tail-associated lysin, an NlpC/P60 peptidase tail-associated lysin, two tail fiber proteins, baseplate assembly protein, baseplate lysozyme, and a tailspike with a pectin lyase domain). The lysis genes include genes for a class II holin (two transmembrane domains in an N-in, C-in topology) and an endolysin (d-alanyl-d-alanine-carboxypeptidase activity) (4). In addition, Spock encodes a SpoIIIE/FtsK homolog, suggesting a requirement for a DNA translocation during the viral infection cycle.

### Nucleotide sequence accession number.

The genome sequence of phage Spock was contributed to GenBank under accession number KF669662.

## References

[1] Ackermann HW Smirnoff WA 1978 Study of lysogeny in *Bacillus thuringiensis* and *B. cereus*. Can. J. Microbiol. 24:818–826 (In French.) 98223

[2] Besemer J Lomsadze A Borodovsky M 2001 GeneMarkS: a self-training method for prediction of gene starts in microbial genomes. Implications for finding sequence motifs in regulatory regions. Nucleic Acids Res. 29:2607–2618 11410670 10.1093/nar/29.12.2607 PMC55746

[3] Green BD Battisti L Koehler TM Thorne CB Ivins BE 1985 Demonstration of a capsule plasmid in *Bacillus anthracis*. Infect. Immun. 49:291–297 3926644 10.1128/iai.49.2.291-297.1985 PMC262013

[4] Wang IN Smith DL Young R 2000 Holins: the protein clocks of bacteriophage infections. Annu. Rev. Microbiol. 54:799–825 11018145 10.1146/annurev.micro.54.1.799

